# Supplementation with nicotinamide limits accelerated aging in affected individuals with cockayne syndrome and restores antioxidant defenses

**DOI:** 10.18632/aging.206160

**Published:** 2024-11-26

**Authors:** Asma Chikhaoui, Kouloud Zayoud, Ichraf Kraoua, Sami Bouchoucha, Anis Tebourbi, Ilhem Turki, Houda Yacoub-Youssef

**Affiliations:** 1 Laboratory of Biomedical Genomics and Oncogenetics, LR16IPT05, Institut Pasteur de Tunis, Université Tunis El Manar, El Manar I, Tunis 1002, Tunisia; 2 Department of Neuropediatrics, National Institute of Neurology Mongi Ben Hamida, Tunis 2092, Tunisia; 3 Orthopedics Department, Béchir Hamza Children’s Hospital, Tunis 2092, Tunisia; 4 Orthopedic and Trauma Surgery Department, Mongi Slim Hospital, La Marsa 2046, Tunisia

**Keywords:** cockayne syndrome, segmental progeroid syndrome, oxidative-stress, niocotinamide, aging

## Abstract

Cockayne syndrome (CS) is a segmental progeroid syndrome characterized by defects in the DNA excision repair pathway, predisposing to neurodegenerative manifestations. It is a rare genetic disorder and an interesting model for studying premature aging. Oxidative stress and autophagy play an important role in the aging process. The study of these two processes in a model of accelerated aging and the means to counteract them would lead to the identification of relevant biomarkers with therapeutic value for healthy aging.

Here we investigated the gene expression profiles of several oxidative stress–related transcripts derived from CS-affected individuals and healthy elderly donors. We also explored the effect of nicotinamide supplementation on several genes related to inflammation and autophagy.

Gene expression analysis revealed alterations in two main pathways. This involves the activation of arachidonic acid metabolism and the repression of the NRF2 pathway in affected individuals with CS. The supplementation with nicotinamide adjusted these abnormalities by enhancing autophagy and decreasing inflammation. Furthermore, CSA/CSB-dependent depletion of the mitochondrial DNA polymerase-γ catalytic subunit (POLG1) was restored following nicotinamide supplementation in CS-affected individuals’ fibroblasts.

This study reveals the link between oxidative stress and accelerated aging in affected individuals with CS and highlights new biomarkers of cellular senescence. However, further analyses are needed to confirm these results, which could not be carried out, mainly due to the unavailability of crucial samples of this rare disease.

## INTRODUCTION

Aging is a process that progressively impacts cell functioning. In the mammalian species, it occurs heterogeneously in multiple organs and tissues, leading to their gradual deterioration. Senescence is defined therefore as a status in which cells are unable to proliferate and, therefore, represent a risk factor for many diseases [1], such as cardiovascular disease [2], dementia [3], and cancer [4] Despite this link with human pathologies, our understanding of the aging process remains limited.

Progeroid syndromes are a group of very rare genetic disorders that exhibit clinical features of pathological aging that occur prematurely. Affected individuals with these diseases share the same characteristics as the elderly, such as hair loss, skin stiffness, neurodegeneration, sensory disorders, etc... It is mainly due to defects in DNA repair pathways [5]. Affected individuals are classified according to the age of onset of clinical manifestations. In infantile forms, affected individuals die at an early age, usually from cardiovascular problems, neurological or muscle degeneration [6]. Fortunately, they remain a category of rare diseases whose global incidence is estimated at around 1:50 000 [7]. Given that segmental progeroid syndromes mimic regular aging, studies of these models have proved very useful not only for identifying the mechanisms involved in the cellular senescence of certain vital tissues but also for studying age-related pathologies [6].

Cockayne syndrome is a rare genetic disorder belonging to the segmental progeroid syndromes; it is caused by genetic variations in *ERCC6* (CS-B form) and *ERCC8* (CS-A form) genes. Both proteins, CSA and CSB, are known to play an essential role in transcription-coupled nucleotide excision repair pathway [8]. CS is a disease of unknown prevalence in Africa. Most affected individuals of African origin were reported in the European cohort, with a total of 17 affected individuals (6 CS-B and 11 CS-A) [9, 10]. Most of them are from North Africa. A few clinical studies describing CS have been reported in the Egyptian [11] and Tunisian populations without genetic characterization [12, 13].

Reactive oxygen and nitrogen species (RONS) are produced by all cells under aerobic conditions and play an important role in senescence and age-related diseases [14]. In fact, the generation of RONS is not only limited to harmful effects on the organism but also contributes to various beneficial processes such as energy production, immune mechanisms, and several signaling pathways. Under normal conditions, cellular ROS may be scavenged by the antioxidant system, restoring redox equilibrium. Nonetheless, cell damage happens when the cell’s antioxidant mechanism fails, either by surpassing its capacity or by becoming less active, which consequently activates the cell death pathway, leading to cellular senescence. Autophagy is a process that degrades damaged cellular components into basic molecules in order to preserve cellular homeostasis and enhance cell survival [15]. Another hallmark of oxidative stress damage is the loss of mitochondrial function and the onset of chronic inflammation. Therefore, substances that directly eliminate ROS or boost cell antioxidant capacity are predicted to help in the preservation of skin homeostasis [16].

Vitamin B3 stands as a known anti-oxidant component as it contributes to improving nerve tissue function, protects against cell damage, and reduces inflammation [17]. It consists of two compounds: nicotinic acid (niacin) and nicotinamide, both of which are distinguished by their pharmacological composition. Vitamin B3 supplementation usually involves nicotinamide, because it is better tolerated within the body, while nicotinic acid is often used to regulate cholesterol levels [18].

Nicotinamide, as an amide water-soluble form, is used in the synthesis of NAD+ which plays a role against oxidative stress and chronic inflammation. Actually, it doesn’t exist any solid proof stating that nicotinamide has distinct molecular targets for skin aging [19].

Oxidative stress is caused by a redox imbalance, which can lead to chronic inflammation. Autophagy is also an important defense mechanism that cells use to respond to oxidative stress. An in-depth study of these processes would lead to the identification of biomarkers for aging.

The aim of our work is to study the expression profiles of genes linked to oxidative stress in CS affected individuals and, in the elderly, and to analyze the effect of nicotinamide in both groups.

## RESULTS

### RT2 profiler gene expression analysis in CS and in healthy elderly donors

**Figure 1:**
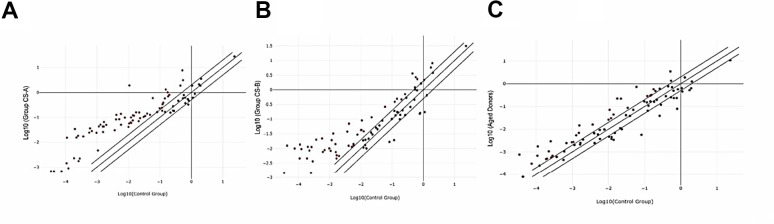
**Scatter plot of differentially expressed genes in the 3 different groups normalized to healthy controls.** (**A**) CS-A, (**B**) CS-B, (**C**) Elderly group.

Oxidative stress-associated gene expression changes in Cockayne syndrome affected individuals with the form A (CS-A) and B (CS-B) as well as in elderly were identified by performing RT2 profiler PCR array. A change in mRNA expression of more than two-fold relative to normal was set as the cutoff value for considering a gene to be under-expressed or over-expressed. Analyses of the data revealed expression changes in 54 genes in the CS-B group, 64 genes in the CS-A group, and 50 genes in the elderly group, among the 84 studied genes that are normalized to healthy young donors (Figure 1).

For the CS-A group, a total of 63 genes exhibited increased expression, while only mitochondrial Peroxiredoxin (*PRDX3)* was under-expressed (fold regulation-2.4)*.* Among the 2 most upregulated genes were Arachidonate 12-lipoxygenase (*ALOX12*) (fold regulation 136) and polynucleotide kinase 3’-phosphatase (*PNKP)* (fold regulation 177).

As for the CS-B group, 49 oxidative stress-related genes were over-expressed such as the albumin gene (*ALB)* (fold regulation 133) and *ALOX12* (fold regulation 122) while two Peroxiredoxin, PRDX3 and PRDX4 were the least expressed with values of -4 and -5 respectively.

In the third group of elderly healthy donors, 33 genes were over-expressed from which the top 2 were also *ALOX12* (Fold regulation 15.85) and the heat shock protein *HSPA1A* (Fold regulation 12.75). In this group, the least expressed genes compared to those in young healthy donors’ fibroblasts were the transcription factor *FOXM1* (Fold regulation -14.32) and *PRDX3* (Fold regulation -10.06).

From the list of differentially expressed genes, statistical significance (P <0.05) was noted only in 5 genes in the CS-B group and in 6 genes in elderly donors’ group. Regarding the CS-A-affected individuals, no statistical significance was noted, however, the same trend was noted in gene expression in the CS-A and the elderly group. (Table 1).

**Table 1:** Significantly altered oxidative stress gene expression in CS-A, CS-B and in elderly compared to young healthy control.

**Symbol**	**Fold change (compared to the young healthy control group)**					
	**CS-A**		**CS-B**		**Old**	
	**Fold change**	**p-value**	**Fold change**	**p-value**	**Fold change**	**p-value**
*ALOX12*	**137.29**	0.349832	**122.41**	**0.033298**	**15.85**	0.346680
*EPHX2*	**8.29**	0.355182	**10.26**	**0.041885**	**4.39**	0.227914
*FOXM1*	1.97	0.390080	**0.20**	**0.015982**	**0.07**	**0.008348**
*GPX7*	0.64	0.747536	1.90	0.239060	**0.42**	**0.042258**
*HSPA1A*	**9.05**	0.269523	1.23	0.555769	**12.76**	**0.005347**
*NQO1*	0.95	0.866789	**4.17**	**0.048009**	**0.33**	0.097544
*PRDX1*	0.71	0.727858	**2.73**	0.196081	**0.15**	**0.034985**
*PRDX3*	**0.41**	0.597151	**0.20**	0.922096	**0.10**	**0.039915**
*PTGS1*	**4.12**	0.361782	**4.22**	**0.042959**	**2.61**	0.368511
*TXNRD1*	**5.70**	0.224758	0.56	0.650882	**3.25**	**0.046888**

**Figure 2:**
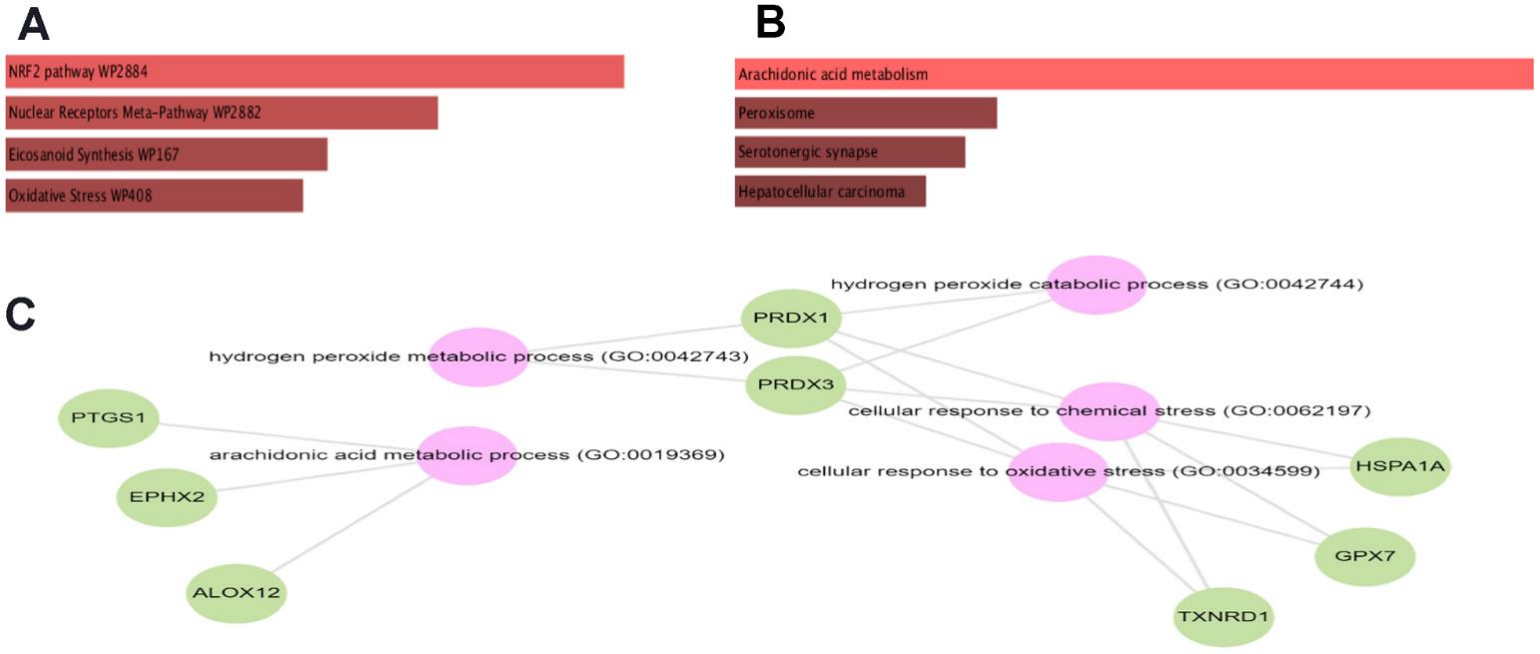
**Bar chart of GSEA obtained from GESalt4 representing.** KEGG (**A**), Wikipathway (**B**) and gene ontology (**C**).

To estimate the functions of identified statistically significant genes, we further undergo gene set enrichment analysis (GSEA) using KEGG and wiki pathway. GSEA analysis showed that the selected set of genes was enriched in various biological processes, cellular components, and molecular functions. The subnetwork shows the following associations from Gene Ontology and from KEGG*: GPX7, ALOX12, EPHX2, and PTGS1* belong to the biological process arachidonic acid metabolic process (GO:0019369) (adjusted P-value= 1.45e-05). While from WikiPathways: The genes *PRDX1, NQO1, TXNRD1, and HSPA1A* are members of the WikiPathway NRF2 pathway (WP2884) (adjusted P-value= 9.57e-04) (Figure 2A, 2B).

### Effects of nicotinamide on oxidative-stress-related gene expression in CS-affected individuals

One of the most well-known pathways involved in oxidative stress is NAD(P)H oxidase pathway. Vitamin B3 is vital for the metabolism of many oxidative-related genes because it provides coenzymes such as NADH and NADPH, which are required for their function including hub genes from the RT2 profiler results such as *PRDX3 FOXM1 NQO1, and ALOX12*.

**Figure 3:**
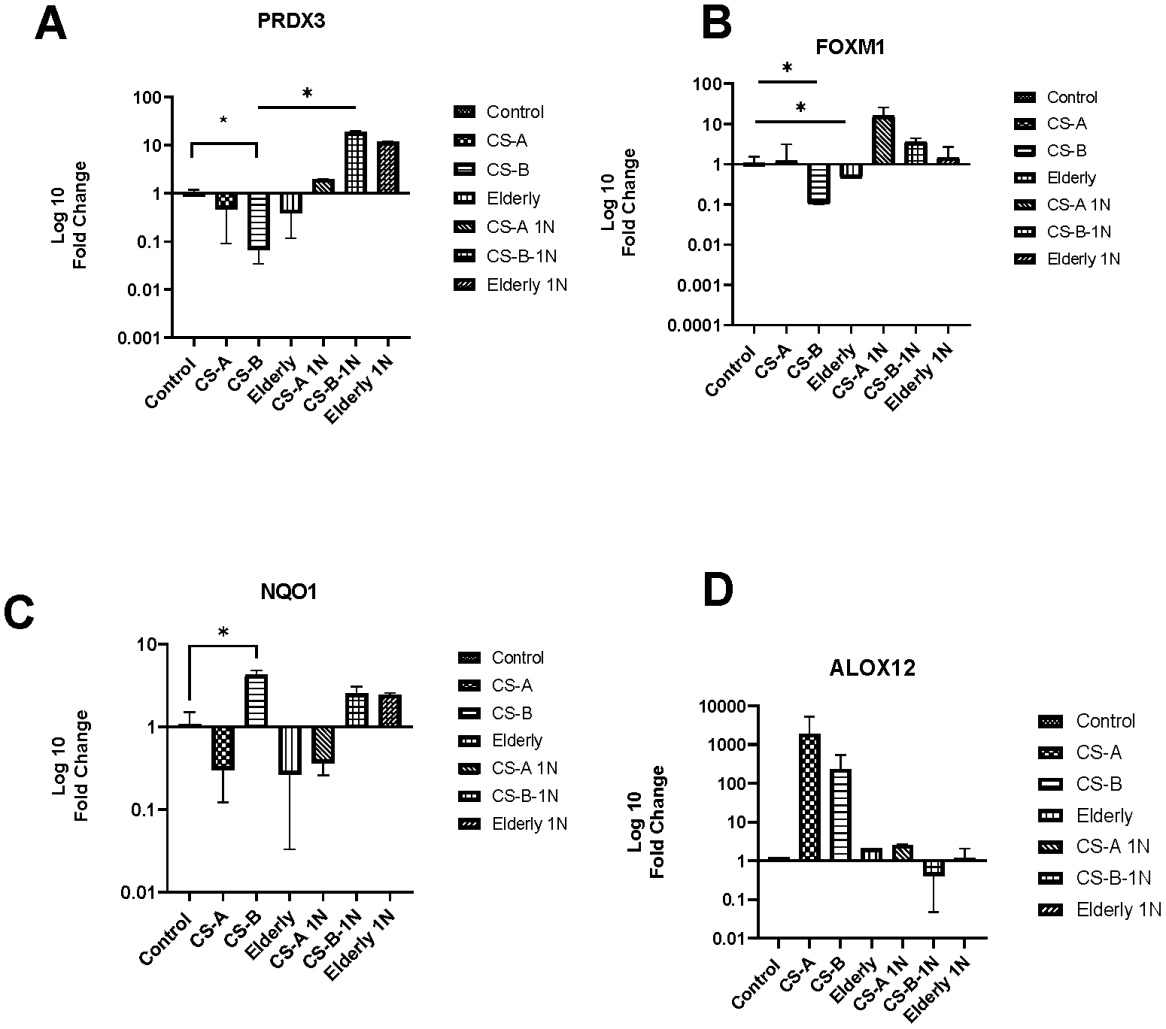
**The level of oxidative stress-related gene expression after nicotinamide stimulation.** The histograms represent fold-changes in genes up-or downregulated in CS-A, CS-B, elderly group and young healthy donors’ group normalized to non-treated control conditions of each group. (**A**) PRDX3, (**B**) FOXM1, (**C**) NQO1 and (**D**) ALOX12.

First, RT-qPCR was performed on this set of genes to validate the results obtained by the PCR array analysis on a larger cohort of affected individuals. Analysis revealed the same tendencies with a significant decrease of *PRDX3 and FOXM1* in CS-B affected individuals with values of fold change respectively (0.06±0.03 p-value =0.009) and (0.1±0.17 p-value =0.04) (Figure 3A, 3B). A significant increase in the transcription level of *NQO1* was also noted in CS-B affected individuals (fold change = 4.23 ±0.6 p-value =0.02) (Figure 3C). *ALOX12* though statistically non-significant maintained higher levels in CS-A and CS-B affected individuals.

Secondly, we examined the effect of nicotinamide (NAM) at a dose of 1mg on their expression levels. We noted that following nicotinamide supplementation, there is an increase in the expression of *PRDX3 and FOXM1* in CS affected individuals and in elderly group with a statistically significant fold change between CS-B and CS-B stimulated with nicotinamide (18,75±1.1 p-value=0.03). This was noted also for FOXM1 (Fold change old 41,99±24, 4,9±1 in CS-B, and 26,33±4 in CS-A). Furthermore, NAM supplementation decreased the expression level of ALOX12 in both groups of CS-affected individuals CS-A and CS-B while it was maintained in the elderly group.

### Effects of nicotinamide on inflammatory-related genes in CS-affected individuals’ fibroblasts

Oxidative stress and subsequent generation of free radicals can harm cell and tissue homeostasis, particularly during aging. We, therefore, explored the effects of nicotinamide supplementation on *P65* and *TNF-a* expression in CS-affected individuals’ fibroblasts. At normal condition, the *P65* gene expression in CS-A affected individuals was 518,49±186, and it declined to 8.87±5, when NAM supplementation was added, to a fold change of 8.87±5.

**Figure 4:**
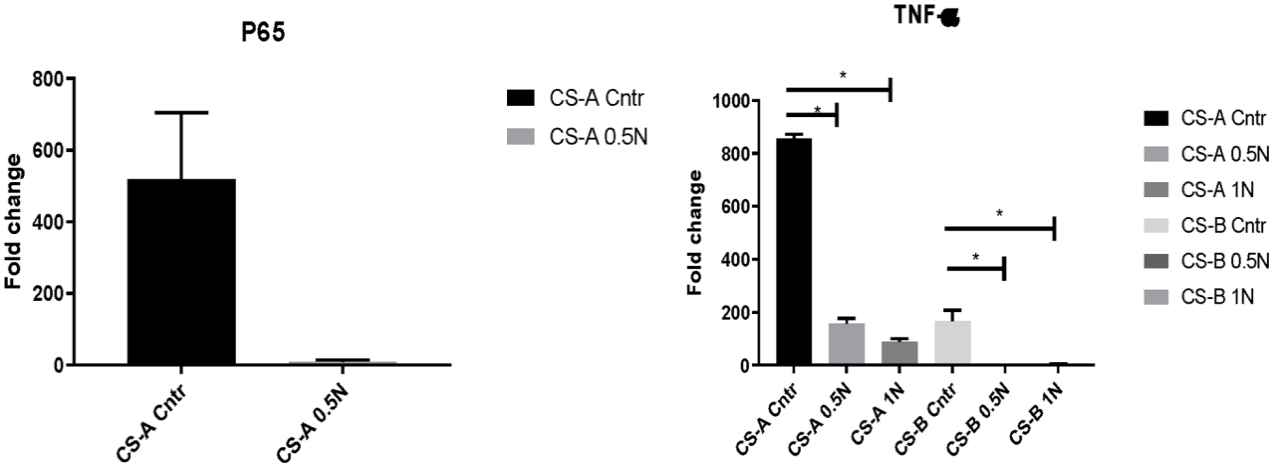
**P65 and TNF-a expression in CS affected individuals’ fibroblast normalized to young healthy donors’ group following of 0.5mg/ml and 1mg/ml NAM supplementation.** (N=1 for CS groups, done in duplicate).

The same tendency was observed in *TNF-a* in CS-A and CS-B affected individuals’ groups that had the fold change of 855,21±16 and 165,80±41 respectively normalized to young control. These levels decreased considerably under different doses of NAM to reach a fold change of 88.7 ±12,57 in CS-A P-value<0.01 and 2.9±1.7 P-value =0.04 in CS-B under a dose of 1mg/ml of NAM (Figure 4). For the elderly group, the results were heterogeneous due to the variability of health conditions and were therefore not presented.

### Effects of nicotinamide on the autophagy-related genes in CS affected individuals’ fibroblasts

The level of *P62* was low in Cockayne syndrome affected individuals (fold change 0.5±0.3) and similar to the elderly group (fold change 0.01±0.08) under starvation conditions. After nicotinamide supplementation for 24h, although not statistically significant the fold change of *P62* gene increased to 14.56± 3.3 in the CS-A group, to 1.2±0.04 in CS-B, and 228±7 in the elderly group.

**Figure 5:**
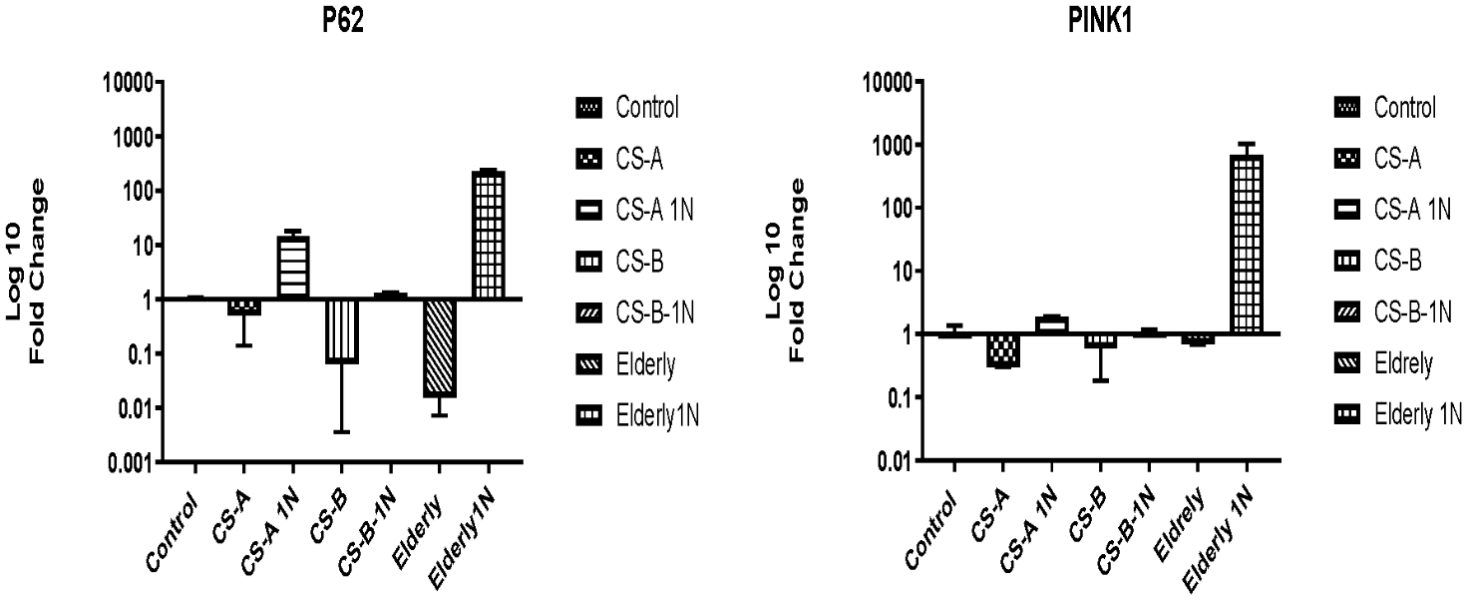
P62 and PINK1 expression in CS-A, CS-B, and elderly affected individuals’ fibroblast normalized to non-treated control conditions of each group under doses of 1mg/ml NAM (N=1 for CS groups done in duplicate).

The same tendency was observed for the *PINK1* gene which was slightly under-expressed in all non-stimulated conditions. NAM stimulation did not significantly affect its expression level, except in the elderly group where its expression increased 687-fold (Figure 5).

### Effects of nicotinamide supplementation on nuclear POLG1 expression in CS-affected individuals’ fibroblasts

**Figure 6:**
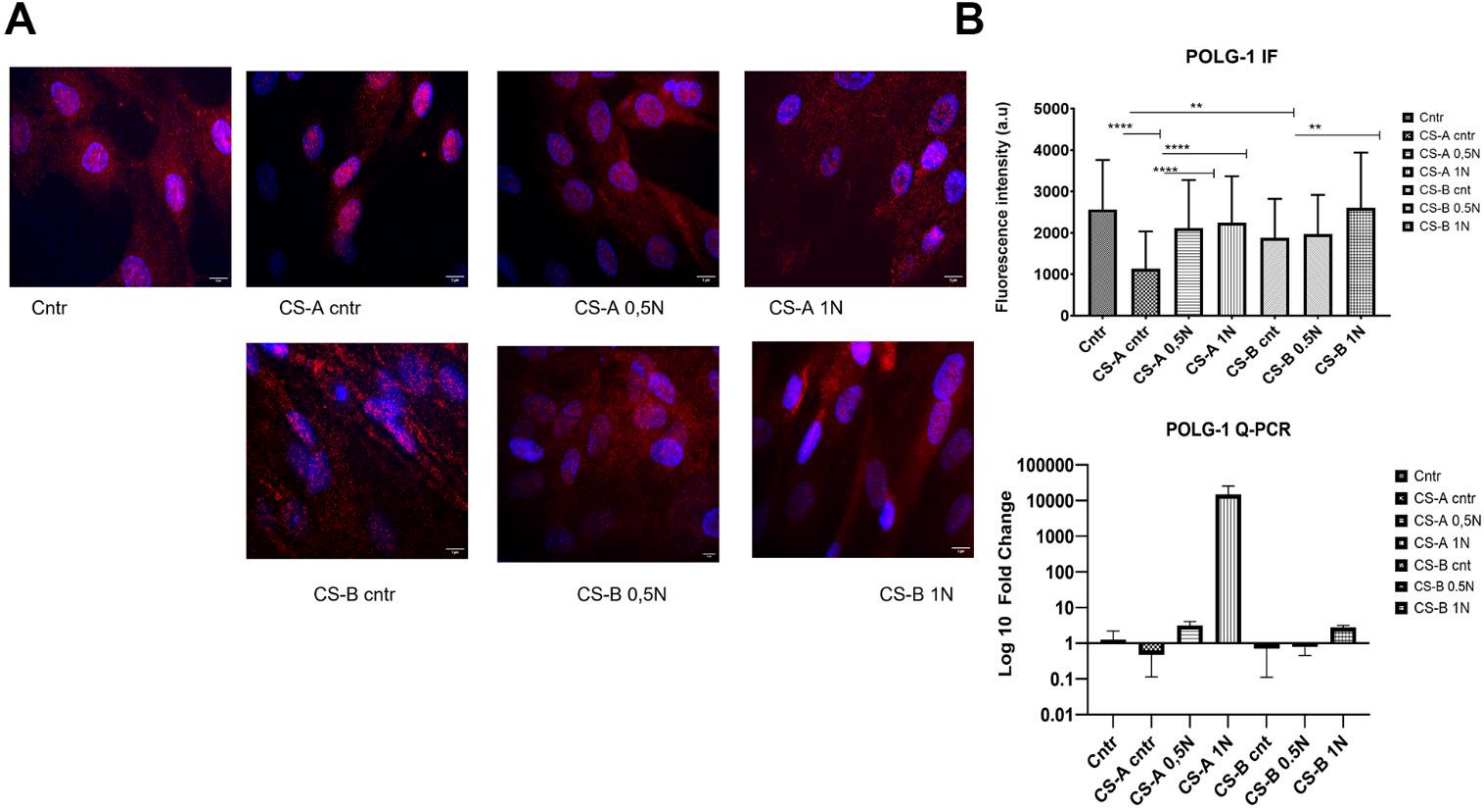
**Level of nuclear POLG 1 upon supplementation with different doses of nicotinamide (NAM).** (**A**) Representative images using a fluorescence microscope after treatment with NAM (0.5 and 1 mg/mL) for 24 h labeled with polg1 (Red) and nuclei with DAPI (blue) (the scale bar is 5 μm). (**B**) corresponding fluorescence intensity and QPCR results for the same gene, the p-values were calculated using unpaired two-tailed Student’s t-test (*p < 0.05, **p < 0.01, ***p < 0.001, ****p<0,0001).

Nuclear POLG1 staining in primary fibroblasts following NAM supplementation at various concentrations for 24 h is shown in Figure 6A. The assessment of fluorescence intensity showed a significant decrease in both CS affected individuals’ forms (CS-A 1137± 800, and CS-B 1883 ±930 compared to the healthy donor control 2566± 1000. All NAM concentrations used for the stimulation could increase the level of polg1 POLG1 in CS affected individuals’ cells as shown by the increased fluorescence intensity (p < 0.05) Q-PCR results, though not statistically significant, showed the same tendency. (Figure 6B).

## DISCUSSION

Age-related diseases represent a public health problem. Identifying dysregulated parameters during aging is, therefore, a major objective of modern medicine. In addition, orphan diseases, of genetic origin, are neglected and are rarely subject to therapy, this is the case of Cockayne syndrome (CS) for which no therapy exists.

Aging is an unavoidable part of life, resulting in a decline of cell functioning. Segmental progeroid syndromes mimic some clinical aspects of normal aging and provide a rare opportunity to understand fundamental processes underlying human aging.

Cockayne syndrome (CS) is a rare genetic disorder predisposing to accelerated aging. Affected individuals suffering from CS are characterized by growth delay, photosensitivity, and neurological manifestations such as impaired motor coordination, speech difficulties, and reduced intellectual capacity. Affected individuals also show signs of premature aging. Cognitive deficiencies may also be observed. The severity of symptoms can vary according to the subtype of the disease [9, 20] In Tunisia, previous work on this syndrome showed clinical particularities and reported 13 affected individuals [21, 22].

Reactive oxygen species ROS are the main factors responsible for skin aging, it contributes to the expression of cyclooxygenase and lipoxygenase, and regulate some inflammatory process [23, 24]. The involvement of oxidative stress in the aging process is therefore well known. In addition, cells taken from affected individuals with CS are known to be hypersensitive to oxidative stress. However, the molecular mechanisms involved remain poorly understood. Previous work has shown that primary fibroblasts derived from affected individuals with CS-A and CS-B forms display an altered redox balance [25, 26] like what we observed in our PCR array results.

In this work, the expression profiles of the two genes related to oxidative stress were different between affected individuals with CS-A or CS-B forms. Indeed, by examining the number of fibroblasts from affected individuals with the CS-A form, we found that they have a moderate response to oxidative stress. These findings are in line with previous work suggesting that cells from CS-A-affected individuals are more sensitive to the action of H2O2 than wild-type cells, while cells from CS-B-affected individuals are even more sensitive [27]. We didn’t find any statistically significant altered gene expression in CS-A group even though it has the same tendency as the elderly group. This may be due to the small number of tested samples via PCR array (N=3) and since one of the tested cases had a different genotype from the others (Table 2).

**Table 2:** List of studied samples.

**Code**	**Age**	**Sex**	**Pathology/genotype**	**Type of experiment/ Cell passage number**
H32	Less than 25	M	Obtained from chirurgical waste during surgeries	P6 (PCR array/QPCR)
H34		M		P5(PCR array/QPCR)
H35		M		P7(PCR array/QPCR)
H36		F		P6(QPCR)
T9		F		P5(QPCR-IF)
T10		M		P5(QPCR)
Old 13	87	M		P7(PCR array/QPCR)
Old14	80	F		P6(PCR array/QPCR)
Old15	89	M		P5(PCR array/QPCR)
Old16	87	M		P8(QPCR)
Old17	85	F		P5(QPCR)
Old20	79	M		P6(QPCR)
CS1	5	F	ERCC8 c.843+1G>C	P6(PCR array/QPCR)
CS2	4	M	ERCC8 c.598_600delinsAA	P5(PCR array/QPCR-IF)
CS6	4	M	ERCC8 c.598_600delinsAA	P5(PCR array/QPCR)
CS11	3	M	ERCC8 c.598_600delinsAA	P5(QPCR)
CS16	8	F	ERCC8 c.598_600delinsAA	P5(QPCR)
CS10	8	M	ERCC6 c.3156dup	P5(PCR array/QPCR-IF)
CS12	7	F	ERCC6 c.3156dup	P5(PCR array/QPCR)
CS14	8	M	ERCC6 c.3156dup	P6(PCR array/QPCR-IF)

The analysis of the expression of the set of genes associated with oxidative stress in elderly in the Tunisian population has never been explored or compared with CS segmental progeroid model as it represents a form of accelerated ageing disorder. With a small number of individuals with rare disorders such as CS, statistical power calculations for the needed sample size in rare disease clinical studies may be impossible [28]. The screening for oxidative stress-related genes in common with regular ageing revealed an increased expression of *ALOX12* in CS-affected individuals. *ALOX12* is often termed plate platelet-type 12-lipoxygenase, codes for an enzyme that oxidizes free fatty acid, crucial for inflammation and oxidative stress defenses [29], it has been therefore implicated in adipogenesis [30]. Its over-expression can promote oxidative stress by triggering low-density lipoprotein oxidation via the p38 mitogen-activated protein kinase (MAPK) and nuclear factor B (NFk-B) pathways [31]. In fact, mice models that mimic CS disorder show clear changes in systemic metabolism during post-natal development, including loss of adiposity which contributes to premature mortality [32].

Within the same rationale, we observed an under-expression of Peroxiredoxin 3 (PRDX3) in both CS affected individuals and elderly. *PRDX3* is a regulator of mitochondrial hydrogen peroxide, presumably scavenging ~90% of the generated ROS [33, 34]. It plays also a role as a putative chaperone under stress conditions [35]. Previous work suggests also that silencing of *PRDX3* would promote oxidative stress–induced cellular senescence [36]. In other disorders with common DNA pathway defects such as in Fanconi anemia, *PRDX3* was found to be downregulated in cellular models which is in line with our findings [37].

As for the molecular signature that was specific to CS-affected individuals, we noted the over-expression of the Polynucleotide Kinase-Phosphatase (*PNKP*) only in the affected individuals with defects in the *ERCC8* gene (CS-A group). The *PNKP* is known to be involved in important DNA repair processes, such as base excision repair [38]. Though *ERCC8* is also implicated in base excision repair pathway, no clear relation between the two genes exists [39, 40] and we speculate that *ERCC8* may play a subsequent pathway role in controlling the *PNKP* expression. We further noted a differential over-expression of the *ALB* (Albumin coding gene) in affected individuals with defects in the *ERCC6* gene (CS-B group). The mRNA levels of Albumin are known to be hepatocyte-specific genes. Its expression in these affected individuals may be related to the hepatic cytolysis observed in these affected individuals [22].

An overall gene set enrichment analysis for the RT2 results highlighted two altered pathways in CS affected individuals namely arachidonic acid metabolism and NRF2 pathway. Arachidonic acid metabolism is a biological process that transforms polyunsaturated fatty acid into different biological compounds via enzymatic reactions mainly Cyclooxygenase, Lipoxygenase, Cytochrome p450, and Anandamide pathways [41]. These processes play a role in regulating inflammatory mediators’ expressions such as TNF-a and enhance the development of senescent cells such as fibroblasts [42]. In fact, in response to DNA damage in CS-affected individuals, it was noted that arachidonic acid-related mediators have an enhanced expression that promotes the immune-response associated with the premature aging phenotype [43].

On the other hand, nuclear factor (erythroid-derived 2)- like 2 (NRF2), controls the genes playing a role against oxidative damage during the process of aging. It is essential for maintaining mitochondrial functioning and controlling inflammatory response by competing with the Nf-kB pathway. NRF2 decreases lead to many disorders including neurodegenerative disorders, and other age-related pathologies [44].

NAD+ is an essential coenzyme that plays an important role in various metabolic pathways, and increasing its rate appears to be a promising strategy for treating a wide variety of pathologies. This has been shown via several studies in animal models and in humans for the treatment of cardiovascular, neurodegenerative, and metabolic disorders [45]. Nicotinamide (NAM) known as vitamin B3 is one of NAD+ precursors that is prescribed usually as a dietary supplement. NAD supplementation has been linked to the treatment of many disorders [46]. It is converted to NAD+ in the last step of the salvage pathway [47].

Recent work has suggested that reduced NAD+ has been observed in CS-affected individuals’ fibroblasts isolated from affected individuals and in mouse models and that this appears to be associated with the aging phenotype [48].

In this study, we observed that NAM enhances the expression of *FOXM1* and *PRDX3* while enhancing *P62*, without having a major effect on *PINK1* in CS affected individuals. Previous studies performed on CS mouse models suggested reduced autophagy [49]. Given the fact that this set of genes are major interactors in the NRF2 pathway, and that previous work suggests that activation of NRF2 helps in maintaining the autophagy pathway through P62 [50]. These results are consistent with previous work suggesting that NAM supplementation restored redox homeostasis via NRF2 and protected cells from oxidative stress, as in the case of aged liver cell models [51] and in Parkinson disease affected individuals’ cells [52]. In addition, NAM supplementation enhances the mitochondrial OXPHOS, which in turn enhances peroxiredoxins’ stability to maintain the stemness of cells through ROS disposal [53, 54].

Further studies suggested that NAD+ increases the activity of *PRDX3*, and *FOXM1* by increasing their acetylation level [55, 56]. It was also proposed that *P62* increases upon stimulation with nicotinamide in the blood of psoriasis-affected individuals [57]. P62 and PINK1 are involved in multiple cellular processes related to aging such as mitophagy [58, 59], oxidative stress response [60], and inflammation [61]. During cell senescence, it was reported that their level declines [62].

In CS affected individuals, the expression of both autophagy-related genes *P62* and *PINK1* was decreased in our cases which is consistent with previous work that showed that in response to stress, CS-B deficient cells demonstrated decreased colocalization of P62 and PINK1 within mitochondria resulting in decreased mitophagy. It is therefore important to note the beneficial role of NAM supplementation [49, 62].

Within NRF2 targeted genes we reported an alteration in the expression of *NQO1* that was specific to CS-B group. *NQO1* is one of the quinone reductases that plays multiple roles in cellular adaptation to stress. One of these functions includes its involvement as a producer of NAD+ for enzymes like PARP and sirtuins [63]. An increased rate of this protein has been noted in the segmental progeroid model of XPG-deficient mice, which highlights its association with the aging process giving the interaction of XPG protein with the core TC-NER factor [64]. Exogenous NAD+ via NAM supplementation does not seem to impact the expression of *NQO1*.

The effect of NAM supplementation was further studied on the altered Arachidonic acid metabolism by examining the expression of *ALOX12* and one of the related inflammatory pathways associated with P56 and TNF-a. Upon administration, we noted the decreased expression of these genes in both CS forms. It is known that nicotinamide prevents lipid peroxidation in experimental animal models possibly including arachidonic acid [65, 66].

Regarding inflammatory genes linked to arachidonic acid metabolism, as to the related inflammatory genes to Arachidonic acid metabolism studies in this work we noted high expression level of TNF-a. In fact, high expression of TNF-α resulted in the premature senescence of human dermal fibroblasts [67].

While most CS affected individuals are known for their photosensitivity, previous work has shown that nicotinamide supplementation improved cell viability loss and attenuated the inflammatory response in photosensitive skin [16] especially through reducing expression level of NF-κB subunit (p50) [68], which was the case of *P50* expression level, noted in CS fibroblasts. This confirms the anti-inflammaging proprieties of this molecule in the CS model.

Further research into the secretory expression of other mediators associated with senescence, such as IL-6 and IL-8, would also have been of interest, given that previous work has suggested that NAM can reduce UV-induced erythema and decrease the production of inflammatory mediators (including cytokines) in the skin [69]. In addition, we have previously shown that the rate of IL-8 was significantly increased in the peripheral blood of the same studied cohort of CS-affected individuals compared to healthy control [70].

Aside from being classified as a form of segmental progeroid disorder, the clinical features of CS-affected individuals share substantial similarities with what is often observed in mitochondrial diseases [71, 72]. Polymerase-γ catalytic subunit (POLG1) is an enzyme that ensures accuracy in the replication and repair of mitochondrial DNA [73]. Mutations in *POLG1* are also associated with several mitochondrial disorders such as Alpers-Huttenlocher syndrome, polyneuropathy, ataxia and progressive external ophthalmoplegia [74, 75]. In a mouse model, mutations in this gene were suggested to be associated with premature aging [76]. It was also shown that in CS cells, the degradation of the POLG1 protein leads to an alteration of mitochondrial functionalities including a reduction in mitochondrial DNA and to oxidative phosphorylation (OXPHOS) [77]. The decrease of *POLG1* expression was further confirmed in this work in both forms (CS-A and CS-B). After NAM supplementation an increase of the level of this polymerase was noted which suggests the activation of an underlying mechanism related to the SIRT pathway [78].

Throughout this work and given the particularity of affected individuals with Cockayne syndrome who do not develop cancer despite the defect in the NER pathway, the detection of biomarkers related to the process of accelerated aging could also represent potential targets for preventing age-related diseases such as cancer, hence the originality of this work.

As has been stated before, the study of genes’ expression in rare disorders could lead to a decrease in the statistical strength of certain analyses, however, it could highlight new biomarkers such as those found in this work [79]. Indeed, as it was recently shown that supplementing NAD+ precursors can restore mitochondrial dysfunction in CS-A or CS-B cells [80]. Here, we support these findings and confirm that nicotinamide would be a key element in future innovative therapeutic strategies that aim to improve the severe phenotype of various DNA repair diseases. However, this study requires further functional analyses to confirm these results, particularly with regard to the exact effect of nicotinamide on the major defects in CS-A/B. These analyses could not be carried out for two main reasons, namely lack of funding and above all the unavailability of certain crucial samples given the rarity of the disease.

## MATERIALS AND METHODS

### Skin biopsy sampling of CS-affected individuals

Eight affected individuals were recruited from the Department of Neuropediatric, National Institute of Neurology Mongi Ben Hamida from 2017 to 2019. Written informed consent was obtained from affected individuals’ families or legal tutors. Skin biopsies were collected for UDS/RRS testing. Three affected individuals were genetically confirmed as CS-B [22] and 5 CS-A [21]. The study was conducted in accordance with the Declaration of Helsinki Principles and approved by Institute Pasteur Ethics Committee in Tunisia (reference 2017/31/I/LR16IPT05/V2), (reference 2018/32/I/ LR16IPT05/V1). Further details are in Table 2.

### Healthy primary dermal fibroblasts

Primary cell cultures of human fibroblasts were isolated from skin biopsies of healthy donors (N=6 young and N=8 old). Small skin fragments have been obtained following surgeries to treat femur fractures in healthy people who have fallen. Further details are in Table 2.

Cells were grown at 37° C in a 5% CO2 humidified atmosphere in Dulbecco's modified Eagle's medium (DMEM) (1g/L glucose) with GLUTAMAX (Life Technologies, Gibco, USA) supplemented with 10% fetal calf serum (Gibco) and 1% penicillin/streptomycin (Gibco). All primary fibroblast cultures were analyzed at similar passages (passages 5 to 7). No anomalies were observed on the morphological aspect, nor on the proliferation rate, as shown in Supplementary Figure 1.

### Nicotinamide supplementation

Cells were seeded in six-well plates and treated with 0.5 mg or 1mg of nicotinamide (Sigma-Aldrich, USA) for 24h in a medium with 0.5 % fetal calf serum (Gibco).

### RNA extraction and reverse transcription

The trizol technique was used to isolate fibroblast RNA. The concentration and purity of RNA were determined using spectrophotometry on a Denovix DS11. Followed by genomic DNA removal using a DNase kit (Invitrogen), starting with an identical amount of 1μg RNA, cDNA synthesis was done whether for PCR array analysis using the RT2 first strand kit (Qiagen) or for further qPCR analyses with SYBR Green (Roche), using the Superscript II Reverse Transcriptase kit and dNTPs primers (Invitrogen) according to the manufacturer's instructions.

### Oxidative stress PCR array

The expression of 84 different genes of oxidative stress was measured by quantitative RT2 Profiler PCR array according to manufacturer recommendation in 3 CS-A, 3 CS-B affected individuals, 3 Old donors and normalized to 3 healthy controls donors (PAHS-065, SABiosciences Qiagen, Germany). The Real-Time PCR reaction was done using the LC480 Roche system. All data were analyzed for relative quantification using the online tool (https://dataanalysis2.qiagen.com/pcr) which automatically calculates the fold-change for the gene expression.

### QPCR analysis

We tested the expression of *ALOX12, PRDX3, FOXM1, NQO1, P62,* and *PINK1* genes using the SYBR Green-based qPCR technique on LC480 light cycler system. Primers were selected from the Primer bank database (https://pga.mgh.harvard.edu/primerbank/). Relative quantification Ct values were obtained from the threshold cycle number of a triplicate test and normalized to the healthy control fibroblast. PPIA and RLPO were used as housekeeping genes. Threshold cycle (Ct) was used to calculate relative gene expression by the 2-ΔΔCT method.

### PolG1 immunofluorescence staining

Cells contained in 8-wells Lab-Tek chamber slide were fixed with 4% paraformaldehyde (PFA) and blocked using blocking buffer containing 5% BSA (Gibco) 0.5% (v/v) TritonTM X-100 (Sigma-Aldrich, USA). Fibroblasts were then incubated with primary antibody solution POLG1 rabbit NBP1-52300 at a dilution of 1/150 overnight at 4° C and further stained with secondary antibody solution Goat Anti-Rabbit (Alexa Fluor® 594) (1:1000 in blocking buffer) for 1 h at room temperature (RT). Coverslips were mounted on slides using ProLongTM diamond antifade mounting medium with DAPI (Invitrogen, USA). Images were captured using Leica DM200 confocal microscope (Leica Microsystems, Wetzlar, Germany) for two CS affected individuals and one control. Quantitative measurements were performed with FIJI software and at least 100 cells were selected.

### Statistical Analyses

All data are expressed as mean ± SEM. Statistical analyses were performed with GraphPad Prism 9 software (GraphPad Software). Significance is set as significant for p-values of less than 0.05.

### Data availability statement

All processed data have been provided in the manuscript. The corresponding author upon reasonable request could provide raw data, generated for this study.

## Supplementary Material

Supplementary Figure 1
