# Supplementary material for: Supplementation with nicotinamide limits accelerated aging in affected individuals with cockayne syndrome and restores antioxidant defenses
Source: Aging (Albany NY). 2024 Nov 26;16(21):13271–87. doi: 10.18632/aging.206160 (PMC11719109; doi:10.18632/aging.206160)
Supplement: Supplementary Figure 1 [file aging-16-206160-s001.pdf]

SUPPLEMENTARY FIGURE

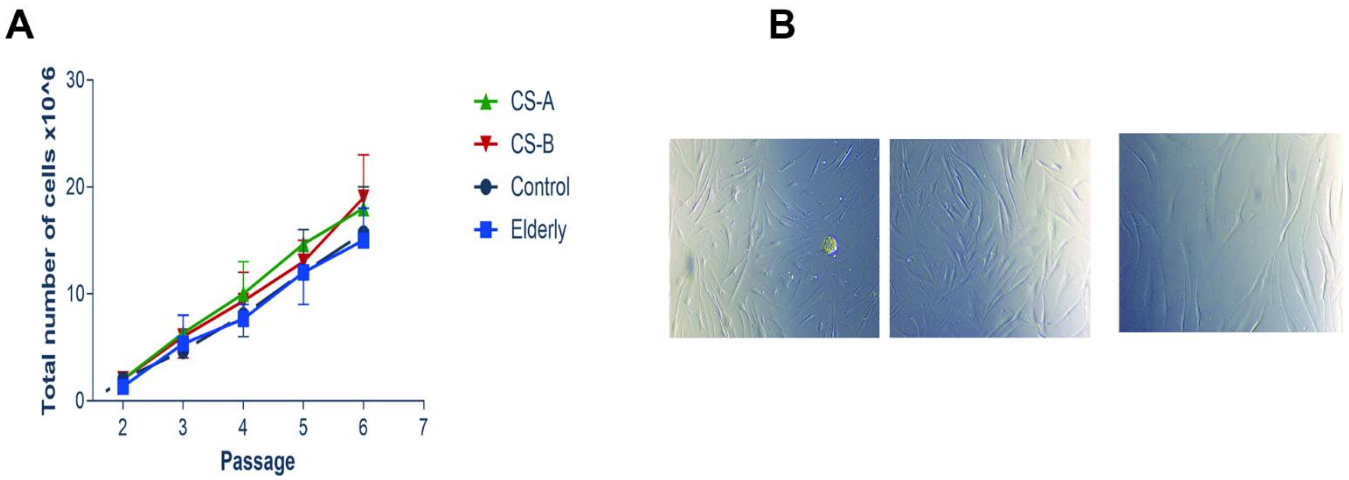

Supplementary Figure 1. CS fibroblasts and morphology (**A**) Skin fibroblasts' proliferation rate. Absolute number of cells from passage 2 to 6 (n = 3). Proliferation was almost similar for CS, elderly, and Healthy donors. (**B**) Morphology of skin fibroblasts of healthy donors and CS-affected individual. Cells were subjected to light microscopy and photographed. A representative image is shown.
